# Childhood obesity prevention in rural settings: background, rationale, and study design of ‘4-Health,’ a parent-only intervention

**DOI:** 10.1186/1471-2458-12-255

**Published:** 2012-04-02

**Authors:** Wesley C Lynch, Jill Martz, Galen Eldridge, Sandra J Bailey, Carrie Benke, Lynn Paul

**Affiliations:** 1 Department of Psychology, Montana State University, Bozeman, MT, 59717-3440, USA; 2 4-H Center for Youth Development, Montana State University Extension, 203 Taylor Hall, Bozeman, MT, 59717-2230, USA; 3 Montana State University Extension, Department of Health and Human Development, 314 Herrick Hall, Bozeman, MT, 59717-2230, USA; 4 Montana State University Extension, Department of Health & Human Development, 316B Herrick Hall, Bozeman, MT, 59717-2230, USA; 5 Montana State University Extension, 314 Herrick Hall, Bozeman, MT, 59717-2230, USA; 6 Montana State University Extension, Department of Health and Human Development, 101 Romney, PO Box 173370, Bozeman, MT, 59717-3370, USA

## Abstract

**Background:**

Childhood obesity in rural communities is a serious but understudied problem. The current experiment aims to assess a wide range of obesity risk factors among rural youth and to offer an 8-month intervention program for parents to reduce obesity risk in their preteen child.

**Methods/Design:**

A two-group, repeated measures design is used to assess the effectiveness of the 4-Health intervention program. Assessments include anthropometric measures, child self-evaluations, parent self-evaluations, and parent evaluations of child. County Extension agents from 21 rural Montana counties recruit approximately 150 parent–child dyads and counties are semi-randomly assigned to the active intervention group (4-Health Educational Program) or a “best-practices” (Healthy Living Information) control group.

**Discussion:**

This study will shed light on the effectiveness of this parent-only intervention strategy in reducing obesity risk factors among rural preteens. The 4-Health program is designed to provide information and skills development for busy rural parents that will increase healthy lifestyles of their preteen children and improve the parents’ ability to intervene effectively in the lives of their families during this critical developmental period.

**Trial registration:**

ClinicalTrials.gov ID: NCT01510587

## Background

Childhood obesity in the US is a major health problem and rates of childhood obesity have increased dramatically over the past 30 years [1]. The fact that obesity rates in rural communities in the US and other countries [2] are as high or higher than those in urban areas is less well-known. For example, rural children had a 54.7% greater risk of obesity than urban children even after controlling for age, gender, race, and socioeconomic status [3]. Given the difficulties of treating obesity, prevention programs are needed that address the unique factors contributing to or helping to prevent child obesity in rural areas.

Childhood and adolescence are developmental periods during which parents have an important influence on a child’s health behaviors and attitudes. Thus, parents play an essential role in guiding the eating habits, weight status, activity levels, and body image of their children [4]. Because parents provide the most important psychosocial influences in the lives of preteen children, working with parents to increase their essential knowledge and skills should strengthen their positive influences, ultimately leading their children to healthier lifestyles, lower obesity risk, and long-term health benefits.

A small number of recent studies have demonstrated that interventions focused on parents can be effective in preventing or treating childhood obesity [5-7]. For example, Project STORY [8-10] is a small feasibility study in which parents and their overweight 8–14 year old children, recruited from rural counties in north Florida, were randomly assigned to one of two treatment groups: parent-only or parent and child, or to a no-treatment (waitlist) control (n ~25/group). Treatment groups received 12 x 90 min intervention sessions delivered over 16 weeks at Cooperative Extension Service offices. Parent-only and parent–child sessions were similar, both focusing on nutritious eating, increased physical activity, and behavior management. Assessments were carried out pre- and post-intervention and at 10-month follow-up. While results indicated that children’s BMI z-scores and energy intake decreased in all three groups, children’s BMIz decreased significantly more in the parent-only group.

While some recent studies have included rural children [10-12], most of these have been feasibility studies not intentionally designed to address the unique problems of rural children and families. Such problems include low income, long travel distances to grocery stores and exercise facilities, few available exercise facilities, seasonal activity options mainly linked to ranching and farming, and limited internet services.

Recognizing these problems, the National Institute of Food and Agriculture (NIFA) in 2008 established a new childhood obesity prevention initiative. NIFA’s Agriculture and Food Research Initiative (AFRI) supports a variety of project types including multi-function integrated research, education, and Extension projects such as the one described here. Project support is mainly targeted to US Land Grant institutions, which operate county Extension offices and state 4-H Youth Development programs. Thus target populations for the AFRI initiatives are rural agricultural communities and families. The purpose of this article is to describe the theoretical background, program components and delivery plan, research design, and methods of the “4-Health” integrated research and outreach project. The name 4-Health reflects the fact that the project is currently focused on children of rural families involved in 4-H programs. The long-range goal of this project is to develop an effective parent-centered child obesity prevention program for rural families.

### Conceptual framework

Intervention program design and assessment plans evolved from an eclectic conceptual framework combining concepts derived from social-cognitive theory [13], the Health at Every Size (HAES) approach [14], and elements of broadly defined social-ecological models [15]. Concepts from the social-cognitive perspective, such as modeling, reinforcement, goal setting, self-regulation, and self-efficacy, guided our thinking during project development and assessment planning. In addition, we adopted core values of the HAES treatment approach [16], including self-acceptance, pleasurable physical activity, and normal eating. We also incorporated ideas from Social Marketing Theory [17], which emphasizes the need for targeting interventions to the needs of specific socioeconomic and cultural groups, and from the family systems perspective [18,19], which emphasizes the need for bi-directional interactive communication between family members. Based on this eclectic approach we envisioned a pathway for child obesity prevention leading from parent education, to parent behavior and attitude change, to parent influence on child and family behaviors, particularly relative to nutrition, physical activity, and body image. Following from this conceptual framework, cognitive and behavioral assessment domains included physical activity and dietary self-reports by parents and children, child behavior assessments by parents, body image and body esteem self-assessments by children, and self-report measures of parents’ perceived self-efficacy at promoting a healthful quality of life for their children and themselves as well as other family members.

### Aims and hypotheses

The research project has three specific aims:

*Develop* an effective parent-centered obesity prevention educational program for rural families that provides parents with new knowledge and skills for healthy living in the areas of nutrition, physical activity, body image, and family communication/parenting. Parents learn how to apply this knowledge within their families and to their preteen children in such a way as to prevent or reduce obesity.

*Implement* the 4-Health Educational program (or a Healthy Living Information control program) over an 8-month period by offering it to parents of 8–12 yo children who are currently participating in Montana’s 4-H Youth Development programs.

*Evaluate* a range of outcome measures gathered from children and parents, which are assessed pre-intervention, post-intervention, and at 6-month follow-up.

Our hypotheses are that:

1. *Children* of parents in the 4-Health Educational program (experimental) group will show significantly greater improvements from pre- to post-intervention in all outcome measures than children of parents in the control group and these improvements will persist or increase at 6-month follow-up.

2. *Parents* in the 4-Health group will show significantly greater improvements from pre- to post-intervention in all outcome measures than parents in the control group and these improvements will persist or increase at 6-month follow-up.

## Methods/Design

During year 1 (May 2009-Apr 2010), intervention programs and assessment plans were developed and focus groups were conducted. Focus groups determined which issues and problems relative to child obesity were of most concern to rural parents and assessed parents’ perceived needs for health information, their interest in program participation, and their time and availability constraints. At the close of year 1, final program design modifications were made, assessment instruments were obtained, assessment plans were finalized, plans for an 8-month pilot study involving parents in six rural Montana (MT) counties were completed, and recruiting of participants for the pilot study began. During year 2 (May 2010-Apr 2011), three of the six pilot counties received the 4-Health Educational program (experimental) and three received the Healthy Living Information program (control). The full implementation of experimental and control programs will begin in 21counties during year 3 (May 2011-Apr 2012). County agents who have a background in Family and Consumer Sciences (FCS) and 4-H (“Agents”) deliver the 4-Health Educational program during face-to-face meetings over an 8-month period between late September and late May. A Food and Nutrition Specialist and the 4-Health Project Director train Agents to deliver the program during one daylong session held in August. All phases of this project were reviewed and approved by the Montana State University Institutional Review Board for the Protection of Human Subjects, FWA 00000165.

### Research design

The original proposal called for a two-group, delayed intervention control (waitlist) design with the experimental group receiving the 4-Health Educational program in year 2 and the waitlist group receiving the program in year 3. However, problems with this design became apparent during year 1. One problem was logistical. With the two-group delayed intervention design, Agents were expected to commit to two years of work during the summer preceding the beginning of the first intervention year. However, since Agents typically develop work plans annually, requiring them to commit to a 2-year project, severely limited the numbers willing to participate. An even more serious problem was the fact that a *no-treatment* (waitlist) control, although quite common in this type of research, would not adequately assess the effectiveness of 4-Health relative to existing prevention approaches. Consultants instead recommended that we use a “best-practices” control group (see below). Finally, as the 4-Health Educational program was being developed, it became clear that several new program activities and delivery methods would require pilot testing. Rather than only test a small portion of the intervention, we decided that a small pilot study was needed in which both the experimental and control programs could be delivered to small groups of participants.

The revised research design is a two-between-groups (experimental v. control) by two-within-groups (pre- v. post-assessment) design comparing the 4-Health Educational (experimental) program to a “best-practices” (control) program. Following additional discussion with consultants, the Healthy Living Information (control) program was devised, which provided participants with written information derived from USDA information sources (see below).

### Semi-random cluster assignment

As an integrated research and outreach project, certain research design compromises had to be made. One compromise resulted from the regional diversity of the Montana population, which consists of a mix of rural and semi-urban counties. Only about half of the state’s population resides in predominantly rural or frontier counties. Since one purpose of the proposed project was to sample widely from these diverse rural populations, it was necessary to select counties for inclusion that were predominantly rural and as widely representative of the rural MT population as possible. A second compromise resulted from the method of participant recruitment. Extension Agents in each county are the point-of-contact between the 4-H Youth Development Programs and parents whose children participate in these programs. For this reason, it was necessary for Agents to have primary responsibility for recruiting qualified participants. However, before Agents could initiate recruiting, the project team felt it was necessary for them to have sufficient information about the treatment groups to inform parents, in general terms, about the nature and extent of their commitment. Furthermore, while some Agents were willing to be randomly assigned to treatment groups, others preferred assignment to only one of these groups.

**Figure 1:**
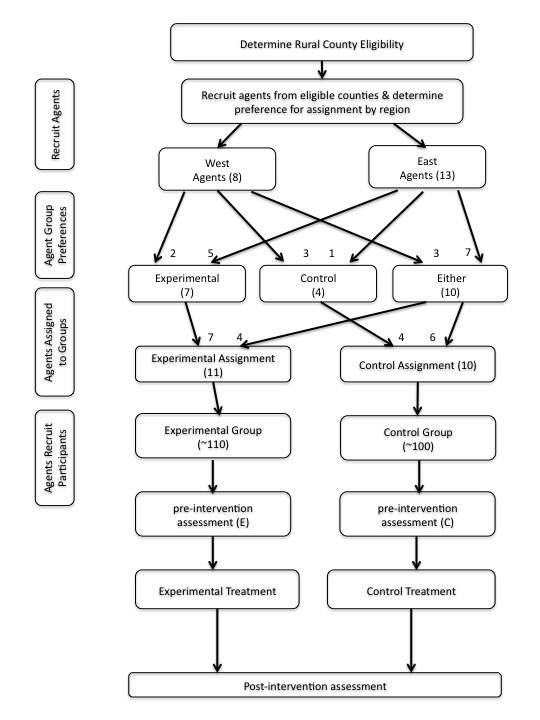
Participant recruitment, assignment, and data collection flow chart.

As a result of these constraints, the research design consists of a semi-random cluster design in which Agents (representing groups of 4-H parents) are first recruited from Eastern and Western regions of the state and their preferences for delivering either the experimental or control program are determined. Agents are then assigned to treatment conditions based on their preference and their geographic location, such that pairs of adjacent counties, with similar participant demographics, are assigned to each treatment condition (Figure 1). This assignment strategy represents a type of regional matching of experimental and control counties. Once assigned to treatment groups, Agents recruit participants within the 4-H clubs in their county or an adjacent county with the aim of recruiting 7–10 parent–child dyads who meet participation criteria. All recruiting materials refer only to the treatment conditions appropriate to the Agent’s group assignment and Agents are asked not to disclose the existence of treatment conditions other than the one for which they are recruiting.

### Marketing and participant recruitment

Agents in rural and frontier MT counties were initially notified of the forthcoming 4-Health Program at the MSU Annual Extension Conference (fall 2010) and tentative interest in participation was subsequently determined. A total of 25 agents representing 30 MT counties expressed an interest and indicated their preference for either the experimental or control groups or their willingness to be assigned. Subsequently, Agents were contacted to determine whether they would be able to recruit a sufficient number of qualified participants. Of the 25 who expressed interest, 21 ultimately agreed to participate. In five cases Agents located in one county agreed to recruit participants from adjacent counties, such that a total of 26 of the 56 MT counties are represented. During a full-day training session Agents received an orientation to the project, learned about marketing, recruitment, and program logistics, reviewed the Parent and Facilitator Guides, and participated in sample program sessions.

Parent recruitment materials describing either the experimental or control programs were provided to all Agents hosting a group in their county. Agents strategically placed recruitment posters and program information in locations frequented by parents of 4-H youth and sent out recruitment materials to 4-H parents in their county. Additional methods used to reach parents were announcements and information at county fairs, announcements in 4-H newsletters, electronic and/or printed announcements to 4-H clubs, emails to 4-H listservs, and phone calls to 4-H leaders. To be eligible for participation parents were required to enroll one child aged 8–12 years old and both parent and child agreed to participate in assessment sessions immediately before and after the 8-month intervention and at 6-month follow-up. Parents selected to participate in the 4-Health Educational (experimental) program also agreed to attend 10 x 90 min face-to-face meetings at or near their County Extension Office. Those parents and children selected for the Healthy Living Information (control) group agreed to attend the pre, post, and follow-up assessment sessions but were only expected to receive and read the printed Healthy Living Information program materials, mailed to their homes in monthly installments over eight months (September – April). Written informed consent for participation in the study was obtained from each parent, who also provided consent for his or her child. Children signed a separate assent form.

### Power estimation

Standard deviation estimates were based on a pilot study consisting of 23 experimental and 16 control participants. Assuming a final participant pool of 75 per group for the current study, with an alpha of .05, and within-groups standard deviation estimates based on the pilot study, power was calculated for two primary outcome variables, BMI z-score change (pre- vs. post-intervention) for children and BMI change for parents. For the first calculation, we assumed a between-groups difference in BMIz for children of 0.15 and a within-groups standard deviation estimate of 0.24. For the second calculation, we assumed a BMI difference of 0.60 and a within-groups standard deviation of 1.18. With 75 participants per group, the estimated power for the child BMIz was 0.96 and the estimated power for the parent BMI was 0.87.

### 4-Health educational (experimental) program development and delivery

Two interventions were developed for this research project, an active intervention (4-Health Educational Program) for use with the experimental group and a “best-practices” (Healthy Living Information) intervention for the control group, described below. Extension specialists from the research team utilized current research, focus group results, and selected components from existing research-based curricula to develop a pilot version of the 4-Health Educational Program curriculum. Steps-to-a-New-You [20] served as the initial curriculum foundation establishing basic health topics including food and nutrition, physical activity, and positive body image. STEPS is part of an on-going program offered through Montana State University Extension, which embodies HAES principles including: valuing health, respecting body-size differences, enjoying the benefits of self-acceptance, enjoying physically active living, and enjoying healthful and pleasurable eating. In addition to the basic health topics, a parenting component was added, which recognized the importance of the child’s psychological and behavioral goals, mutual respect between parent and child, and appropriate parental encouragement via positive reinforcement. This component also emphasizes effective communication, such as listening and responding to the child’s feelings, rather than lecturing and criticizing, resulting in more effective parent–child interaction. Parents are taught how to motivate and help support their children, thereby encouraging positive changes in behavior. This parenting component draws heavily from the Food-Related Intergenerational Discussion Group Experiences (FRIDGE) curriculum, a family-centered program based on a grounded theory approach and designed to improve family communication and decision-making in relation to nutrition [21]. Finally, the 4-Health Educational curriculum incorporated components of *Essential Elements for Positive Youth Development*[22], a curriculum for adults which draws on the developmental theory of Lerner et al. [23], emphasizing the need to align youth needs with available environmental assets.

Thus, the 4-Health Educational program includes the topics, objectives, and activities outlined in Table 1. *Food and Nutrition:* choose foods and beverages packed with nutrients; increase fruits and vegetables, low-fat dairy foods and beverages, and whole grain choices; decrease sweetened beverage consumption; eat meals and snacks regularly; choose food portions appropriate for activity level; increase times when families eat together; practice the principles of normal, healthy eating; avoid unhealthy weight-control practices. *Physical Activity:* create an accessible physical environment that promotes an active lifestyle; reduce sedentary time; promote physical activity via whole-family communication; take advantage of available community sites for physical activity. *Positive Body Image:* encourage size and body acceptance of self and others; understand media and environmental influences on the development of body image; teach and model healthy self-esteem, respect, and confidence. *Parenting*: practice good communication skills; provide high levels of love, warmth, and boundaries; advocate for preteen child; provide opportunities for preteen child to grow and develop his or her own identity.

**Table 1:** 4-Health Educational Program Sessions

**Topic**	**Participant Objectives**	**Activities**
Program Introduction & Focus Areas	· Learn about the program focus areas and their related behaviors: Food and Nutrition, Physical Activity, Body Image, and Active Parenting	· Program focus areas and motivation activity · Active Parenting introduction and Worksheet · Introduction to website and participant/preteen home cooking assignments
Parenting Styles & Food and Nutrition Basics	· Explore different parenting styles and how they support preteen health · Learn about nutrition basics (MyPlate and nutrient-rich foods) · Create SMART Plans for each program focus area	· Parenting Styles introduction and activity · Basics of nutrition presentation · SMART Plan introduction and goal setting activity · Take-home food and nutrition environment assessment
Stages of Child Development & Physical Activity Basics	· Compare the stages of child development and how to effectively parent the preteen stage · Learn about physical activity basics for preteens · Adapt and continue with SMART Planning	· Stages of child development information and activity · Basics of physical activity presentation · SMART Planning follow-up, additions, and adaptations · Take-home physical activity environment assessment
Family Communication & Mealtime	· Learn about effective family communication and the FRIDGE curriculum · Consider current family mealtime practices and discuss how best to enhance them · Adapt and continue with SMART Planning	· Family communication and FRIDGE curriculum information and discussion · Dinnertime activity · SMART Planning follow-up, additions, and adaptations · Take-home grocery store scavenger hunt
Beyond the Basics of Food and Nutrition	· Learn about and discuss further food and nutrition topics · Consider how portion choices have changed over time and how we make decisions about serving size · Adapt and continue with SMART Planning	· Beyond the basics of nutrition information and breakout discussions · Portion distortion presentation and activity · SMART Planning follow up, additions, and adaptations · Take-home internet recipe activity
Body Image – The Basics & Beyond	· Learn about body image and discuss situations that affect preteens · Understand the impact of media messages on preteens and how to proactively address the messages · Adapt and continue with SMART Planning	· Minimize media impact activity · Body image information and discussion · SMART Planning follow up, additions, and adaptations · Take-home body image environment assessment
Beyond the Basics of Physical Activity	· Learn about and discuss further physical activity topics · Consider motivations and barriers to physical activity in their family · Adapt and continue with SMART Planning	· Motivations and barriers activity and worksheet · 100-Calorie health information and activity · SMART Planning follow up, additions, and adaptations · Take-home family physical activity worksheets
Moving into the Teen Years & Food and Nutrition Skill-Builders	· Learn tips and techniques to help preteens make a healthy transition into teen years · Apply the concepts of satiety and energy density to family food choices · Learn how to help preteens use the nutrition food label · Adapt and continue with SMART Planning	· Moving into the teen years information and discussion · Satiety and energy density information and activity · Nutrition Tool: Label reading activity · SMART Planning follow up, additions, and adaptations · Take-home label reading and hunger and satiety activities
Parents as Community Change Agents & Physical Activity and Body Image Skill-Builders	· Examine communities and what parents could do to promote healthy changes · Use scenarios and discussion to determine how best to address body image issues with preteens · Participate in a circuit course activity · Adapt and continue with SMART Planning	· Community change activity · Body image how-to’s activity · Circuit training exercise and take-home activity · SMART Planning follow up, additions, and adaptations · Take-home preteen community change worksheet
Moving Forward with Healthy Habits	· Reflect on the progress made throughout the program · Learn strategies to keep up with behavior changes made throughout the program · Plan for continuation of SMART Planning	· Healthy preteens reflection activity · Moving forward with healthy habits presentation · Keeping up with SMART Planning information and activity · Final program evaluation

Facilitator guides include all 4-Health lessons plans, agendas, scripted PowerPoint presentations, handouts, a resource list, participant and facilitator evaluations, and take-home activities, which establish and apply skills for lifestyle behavior changes for youth and parents. Each lesson includes one or two objectives that cover health topics (food and nutrition, physical activity, body image), but also integrates objectives that introduce social-cognitive strategies, such as SMART Planning [24], a goal setting method that helps users identify what they want to accomplish and assists them in creating a plan to reach their goals related to each health topic. A website developed for the general public, with a protected user-only section, allows forum discussions among participants.

### Healthy living information (control) program

The Healthy Living Information program consisted of written information based on the USDA’s ChooseMyPlate [25] website and the American Dietetic Association’s [26] internet resources. Approximately every three weeks between late September 2011 and April 2012, corresponding approximately to the meeting times for the 4-Health Educational program, participants in the control group receive mailed packets of information. Table 2 is a list of the packets and topics of the materials sent in each of 10 mailings.

**Table 2:** Healthy Living Information Program Packets and Topics

**Packet**	**Topic**
1	**Binder, welcome letter, and introduction to MyPlate brochure:** Build a healthy plate Cut back on foods high in solid fats, added sugars, and salt Eat the right amount of calories for you Be physically active your way
2	**MyPlate “Ten Tips” Handouts:** Choose MyPlate Add More Vegetables to Your Day Focus on Fruits Healthy Eating for Vegetarians Be a Healthy Role Model for Children Cut Back on Your Kid’s Sweet Treats Make Half Your Grains Whole Salt and Sodium
3	**Focus on Vegetables:** What’s in the Vegetable Group? How much is needed? What counts as a cup? Health benefits and nutrients Tips to help you eat vegetables
4	**Focus on Fruits:** What’s in the Fruit Group? How much is needed? What counts as a cup? Health benefits and nutrients Tips to help you eat fruits
5	**Focus on Dairy:** What’s in the Dairy Group? How much is needed? What counts as a cup? Health benefits and nutrients Tips to for making wise choices in the dairy group
6	**Focus on Grains:** What’s in the Grains Group? How much is needed? What counts as an ounce? Health benefits and nutrients Tips to help you eat whole grains
7	**Focus on Protein:** What’s in the Protein Group? How much is needed? What counts as an ounce? Nutrients and health implications Vegetarian choices Tips to help you make wise choices from the protein foods group
8	**Focus on Oils:** What are “oils”? How are oils different from solid fats? Why is it important to consume oils?
9	**Focus on Empty Calories:** What are “empty calories”? What are “solid fats”? What are “added sugars”?
10	**Focus on Physical Activity and Body Image:** What is physical activity? Why is it important? How much is needed? How many calories are used? Tips for increasing physical activity Tips for talking to your child about body image

### Assessment topics and procedures

Before beginning of the program, parent participants and one of their 8–12 year old children attend the pre-program assessment session. At this session, weight, height, blood pressure, and heart rate are taken from each parent and child. Parents and children also complete separate survey packets (see below). All participants complete identical assessments again after completing the intervention programs and at a 6-month follow-up. All assessments are carried out with groups of participants at county Extension offices (or nearby locations). Specific assessment times generally occur during the three weeks preceding (pre) or following (post) program delivery and within two weeks of the 6-month follow-up period. Teams of data collectors (3–4 members per team), hired and trained for each data collection period, travel to each county data collection site at designated times. Data collectors receive two days of intense training on the research protocol, use of equipment, and how to interact with the program participants so that data collection will be highly consistent and sensitive to participants.

Participants in both the experimental and control groups receive $50 for completing each assessment session. Participants who complete the study also receive a $200 stipend. In order to receive the program stipend, participants in the 4-Health Educational Program (experimental) group are required to attend at least eight out of the 10 face-to-face sessions. Experimental program participants also receive a small travel stipend depending on the distance traveled to and from sessions and the number of sessions attended.

#### Anthropometric measures

The following measures are obtained both from parent and child: height, weight, resting heart rate, and resting systolic and diastolic blood pressure. Height is measured in centimeters two times using a Seca 217 stadiometer and weight is measured in pounds, using a Tanita WB-110A scale, both procedures following the National Health and Nutrition Examination Survey (NHANES) protocol [27]. Three independent measurements are taken for resting heart rate and resting blood pressure, with one minute of quiet rest between each measurement, using an automated Omron HEM-705-CP monitor and following the Omron manual protocol.

#### Child self-evaluations

Children complete the following questionnaires concerning their attitudes and behaviors related to physical activity, food and nutrition, body image, appearance, body esteem, self-esteem, and quality of life.

##### Physical activity/healthy foods efficacy scale for children

Self-assessment of goal setting for physical activity and healthy food choices and self-efficacy for physical activity and healthy food choices [28].

##### Physical activity questionnaire for older children

Self-report of physical activity in children 8–14 years of age [29].

##### Youth risk behavior survey

Self-assessment of health-risk behaviors among youth. We include only the items concerning physical activity and unhealthy dietary behaviors [30].

##### Coordinated approach to child health-health behavior questionnaire, section D

Self-assessment of behavior and psychosocial variables related to nutrition and physical activity [31]. We include only items about children choosing and fixing their own snacks and meals.

##### Family meals

Self-assessment of priority, atmosphere, and structure/rules at family meals [32].

##### Child body image scale

Self-assessment of body size perception and body size dissatisfaction [33]. Seven gender-specific body shape figures are presented representing standard percentile BMI differences for healthy children. Children are asked to: “identify the body figure most like your own” and “identify the body figure you would most like to have.” Body size perception is calculated as the difference between the BMIz representing the shape selected as *‘most like your own’* and the child’s *actual* BMIz. Body size dissatisfaction is calculated as the difference between the BMIz representing the shape selected as *‘would most like to have’* and the child’s *perceived* BMIz.

##### Body Esteem Scale (BES)

Self-evaluation of own body and appearance [34]. The BES has three subscales: BE-Appearance (general feelings about appearance), BE-Weight (weight satisfaction), and BE-Attribution (evaluations attributed to others about one’s body and appearance).

##### Socio-cultural attitudes toward appearance questionnaire

Measures endorsement of societal appearance ideals [35].

##### Coopersmith self-esteem inventory

Assesses attitudes toward self in personal, social, family, and academic areas of experience [36].

##### Kidscreen-27

Self-assessment of child’s quality of life, including physical well-being, psychological well-being, parent relations and autonomy, social support and peers, and school environment [37].

#### Parent self-evaluations

Parents complete the following questionnaires concerning their attitudes and behaviors related to physical activity, food and nutrition, body image, personal appearance, parenting style, family communication, making healthy choices for their family, and various demographic characteristics.

##### Exercise self-efficacy scale

Assesses self-efficacy expectations related to the ability to continue exercising in the face of barriers [38].

##### Physical activity

Assesses leisure time physical activity [39]. We add questions about additional activity throughout the day.

##### Health-promoting lifestyles profile II

Evaluates various dimensions of health-promoting lifestyle [40]. We include only items defining three of the dimensions: nutrition, physical activity, and stress management.

##### Child feeding questionnaire

Self-assessment of parental beliefs, attitudes, and practices regarding child feeding, including perceived responsibility for child feeding, parent perceived weight, perceived child weight, parents’ concerns about child weight, monitoring of child’s food intake, and restriction of child’s food intake and pressure to eat [41].

##### Family meals

Same as child questionnaire (see above).

##### NHANES flexible consumer behavior module, 2009–2010

Includes selected items concerning overall diet quality, frequency of eating away from home, and frequency of eating at fast food restaurants [42].

##### NHANES consumer behavior family questionnaire, 2009–2010

Includes selected items concerning availability in the home of the following foods: fruit, dark green vegetables, salty snacks, skim milk, and soft drinks [43].

##### Appearance schemas inventory-revised

Consists of two subscales in relation to one’s cognitive-behavioral investment in appearance. Self-evaluative salience assesses the extent to which individuals define or measure themselves and their self-worth by their physical appearance, which they deem influential in their social and emotional experiences. Motivational salience assesses to the extent to which persons attend to their appearance and engage in appearance-management behaviors [44].

##### Parenting style and dimensions questionnaire

Three-factor self-assessment of parenting style. Authoritative parenting style items evaluate parent–child warmth and connection, parental use of reasoning, inductive parenting, and autonomy granting. Authoritarian parenting style items evaluate physical coercion, verbal hostility, and non-reasoning/punitive disciplinary practices. Permissive parenting style items evaluate parental indulgence and inconsistency [45].

##### Family communication scale

Self-assessment of positive aspects of family communication, focusing on a free flowing exchange of information both factual and emotional. Assesses the degree to which family members feel unconstrained and satisfied with the communication within their family [46].

##### Healthy choices self-efficacy

Assesses parental confidence in making overweight-related behavior changes for their family [47].

##### Demographic

Items include race/ethnicity of parent and child, marital status, employment, education, income, and distance from home to the nearest convenience grocery store, the nearest full service grocery store, and the child’s school.

#### Parent evaluation of child

Parents complete the following questionnaires on obesity related topics as they relate to their child including the child’s sedentary behavior, food intake, and quality of life.

##### Sedentary behavior questionnaire

Assesses a broad range of sedentary activities common among adolescents [48].

##### Fred Hutchison cancer research center food frequency questionnaire

Quantitative assessment of child’s nutrient intake. Detailed nutritional analysis of the child’s diet is derived by standardized procedures developed by the Nutrition Assessment Shared Resource [49].

##### Kidscreen-27

Same as child questionnaire (above) but completed by the parent as the child’s proxy.

##### Other information

Parents provide information on the child’s usual week day and weekend sleep and wake times, frequency of family dinner consumption, and frequency of the child’s breakfast eating.

## Discussion

Review of recent literature suggests that parents can have a significant impact on the prevention of childhood obesity and that parent-only interventions may be more effective than child-only interventions or those involving both parent and child.

Although it is known that rural children are at greater risk for obesity than their urban peers [3], previous studies involving rural families have been limited in scope and not specifically designed with a focus on the special needs, interests, benefits, and limitations facing rural families. Rural parents face a number of unique challenges in their attempts to maintain healthy living environments for their children. For example, fresh fruits and vegetables are less readily available in rural communities; major grocery stores, exercise facilities, hospitals or clinics, schools and even neighbors are often located at great distances from their homes; many rural families have limited or seasonal incomes and limited access to modern high-speed communications. Thus any effective obesity prevention program must be developed with these limitations clearly in mind.

One additional consideration when developing an obesity prevention program for rural families is that effective programs demand a unique delivery method. Extension agents with Family and Consumer Science and 4-H experience are particularly well suited to serve as program trainers in rural communities for several reasons. First, they live in these rural communities and are intimately familiar with their residents, including their needs, interests, and the limitations such as those noted above. Second, they have extensive experience in the development and delivery of health programs. Third, as university faculty members, they recognize the importance of research and the need to gather objective data that can demonstrate program effectiveness. Finally, because they are involved in 4-H programs, they are intimately familiar with the individual families and children involved in these programs.

Given the above considerations and guided by concepts derived from social cognitive theory [13], principles of HAES [45], and knowledge gained from programs such as STEPS [20], FRIDGE [21], and the 4-H Council’s *Essential Elements for Positive Youth Development*[22]*,* we developed a unique parent-only intervention designed specifically for rural and frontier families in Montana. By developing our program specifically for parents involved in a large ongoing youth development program (i.e., 4-H Youth Development Program, sponsored by the USDA), our hope is that this intervention can potentially be applied to other public and private youth development programs, such as Boy Scouts and Girl Scouts or programs offered by religious organizations in which parents and their children both actively participate.

Parents of young children are in a unique position to impact the health behaviors of themselves, their children, and their families. Beyond the family, well-educated parents can extend their influence into their children’s schools and into their larger communities. As active members of community social, political, and religious groups parents have the potential to limit and help reduce the epidemic of obesity.

## Competing interests

The authors have no competing interests.

## Authors’ contributions

WL is coPI responsible for research design and methods issues; he contributed to the selection of assessment instruments and developed the current manuscript. He also contributed to development of the intervention programs and focus group evaluation. JM is coPI responsible for coordination with 4-H Extension agents and with 4-H families. She played a major role in recruitment of participants and in development of the 4-Health Educational Program session topics. GE is the research associate primarily responsible for selection of assessment instruments and obtaining of approvals for their use. She has also been centrally involved in assessment planning and preliminary data collection and analysis. SB is the primary consultant on parenting and family issues and played a key role in the development of program modules dealing with parenting and family communication issues. CB is the Project Director responsible for program development and project management. LP is coPI on the NIFA grant supporting this project and took the lead role in the development of the 4-Health Educational Program and selecting topics for the Healthy Living Information Program. All authors read and approved the final manuscript.

## Pre-publication history

The pre-publication history for this paper can be accessed here:

http://www.biomedcentral.com/1471-2458/12/255/prepub
